# Modification of the effect of ambient air temperature on cardiovascular and respiratory mortality by air pollution in Ahvaz, Iran

**DOI:** 10.4178/epih.e2020053

**Published:** 2020-07-18

**Authors:** Sohrab Iranpour, Soheila Khodakarim, Abbas Shahsavani, Ardeshir Khosravi, Koorosh Etemad

**Affiliations:** 1 Department of Epidemiology, School of Public Health and Safety, Shahid Beheshti University of Medical Sciences, Tehran, Iran; 2 Department of Environmental Health Engineering, School of Public Health and Safety, Shahid Beheshti University of Medical Sciences, Tehran, Iran; 3 Environmental and Occupational Hazards Control Research Center, Shahid Beheshti University of Medical Sciences, Tehran, Iran; 4 Iranian Ministry of Health and Medical Education, Tehran, Iran

**Keywords:** Temperature, Air pollution, Cardiovascular diseases, Respiratory tract diseases, Effect modification

## Abstract

**OBJECTIVES:**

This study investigated the modification of temperature effects on cardiovascular and respiratory mortality by air pollutants (particulate matter less than 2.5 and 10 µm in diameter [respectively], ozone, nitrogen dioxide, carbon monoxide, and sulfur dioxide).

**METHODS:**

Poisson additive models with a penalized distributed lag non-linear model were used to assess the association of air temperature with the daily number of deaths from cardiovascular and respiratory diseases in Ahvaz, Iran from March 21, 2014 to March 20, 2018, controlling for day of the week, holidays, relative humidity, wind speed, air pollutants, and seasonal and long-term trends. Subgroup analyses were conducted to evaluate the effect modification for sex and age group. To assess the modification of air pollutants on temperature effects, the level of each pollutant was categorized as either greater than the median value or less than/equal to the median value.

**RESULTS:**

We found no significant associations between temperature and cardiovascular and respiratory mortality. In the subgroup analyses, however, high temperatures were significantly associated with an increased risk of cardiovascular mortality among those 75 years old and older, with the strongest effect observed on day 0 relative to exposure. The results revealed a lack of interactive effects between temperature and air pollutants on cardiovascular and respiratory mortality.

**CONCLUSIONS:**

A weak but significant association was found between high temperature and cardiovascular mortality, but only in elderly people. Air pollution did not significantly modify the effect of ambient temperature on cardiovascular and respiratory mortality.

## INTRODUCTION

Among the causes of mortality, cardiovascular diseases (CVD) and respiratory diseases are the most strongly associated with temperature [1,2]. Because CVD and respiratory diseases are the most common causes of death worldwide [3], investigation of this association is of great importance. In previous studies, a non-linear association (J-, W-, or U-shaped) between temperature and mortality has been reported [4]. A delayed effect of temperature on mortality has also been established; that is, temperature can affect mortality not only on the same day as exposure, but also in subsequent days or weeks [5]. Therefore, to investigate the association between temperature and mortality, a model is required that takes into account both non-linearity and delayed effects. In 2010, Gasparrini et al. [6] proposed the distributed lag non-linear model (DLNM), a relatively flexible model that simultaneously takes into account non-linearity and delayed effects. The DLNM was then extended to form a framework termed the penalized DLNM in 2017 [7]. With this model, it is also possible to calculate the cumulative effects [4,6,8].

In addition to temperature, air pollutants are well-known to impact cardiovascular and respiratory mortality and can act as confounders or effect modifiers of the temperature-mortality relationship [9]. However, in some studies they have been ignored [10-12] or considered only as confounders [13]. Given the frequent simultaneous exposure to ambient temperature and air pollution, one can expect to see the synergistic effects of these factors in human physiology [9], and the evaluation of the interaction (or effect modification) between temperature and air pollution is of great importance, especially in developing countries. The limited number of studies conducted on this topic have yielded inconsistent results. Li et al. [14] reported on this interaction for CVD, but not for respiratory disease. In a study of CVD, Chen et al. [15] found no significant effect modification by particulate matter less than 2.5 µm and 10 µm in diameter (PM_2.5_ and PM_10_, respectively) or ozone (O_3_). In another study, conducted by Lokys et al. [16], no interaction was found for either CVD or respiratory disease.

The association between temperature and mortality may be greatly altered by factors such as population, climate, and geographic characteristics [17-19], indicating the need to investigate this association in different regions to better understand how to reduce mortality. However, the majority of previous studies have been conducted in developed countries, mainly in the United States [20-22] and Europe [15,23,24], and less is known about this problem in developing countries, particularly those in the eastern Mediterranean and Middle East, which are among the most vulnerable areas to climate change [25]. To the best of our knowledge, these interactions have not been investigated in Iran, and some studies that have been conducted on the relationship between temperature and mortality have not considered the confounding effects of pollutants [10-12], non-linearity, or the delayed effects of temperature on cardiovascular and respiratory mortality [11,12,26].

In the current study, we investigated the association of temperature with cardiovascular and respiratory mortality and effect modification by air pollutants (nitrogen dioxide [NO_2_], carbon monoxide [CO], sulfur dioxide [SO_2_], PM_10_, PM_2.5_, and O_3_) in Ahvaz, Iran using a generalized additive model combined with the penalized DLNM.

## MATERIALS AND METHODS

### Mortality, weather, and air pollution data

In this study, we used the daily counts of deaths from CVD (International Classification of Disease, 10th revision [1CD-10] codes I00-I99) and respiratory diseases (ICD-10 codes J00-J99) supplied by the Municipal Center of Disease Control and Prevention among residents living in the city of Ahvaz, Iran between March 21, 2014 and March 20, 2018. Ahvaz, the capital of Khuzestan Province, is located in southwestern Iran (31°32′ N and 48°68′ E) [27].

Meteorological data, including daily mean temperature, wind speed (m/s), and relative humidity (%), within the same period were obtained from the Iranian Meteorological Data Sharing Service System (https://data.irimo.ir/).

Measurements of hourly concentrations of PM_2.5_ (µg/m^3^), PM_10_ (µg/m^3^), O_3_ (µg/m^3^), NO_2_ (µg/m^3^), CO (µg/m^3^), and SO_2_ (µg/m^3^), recorded at all 4 air quality monitoring stations during the study period, were obtained from the Environmental Protection Agency (EPA). For each station, maximum 8-hour moving averages for CO and O_3_, the maximum 1-hour value for NO_2_, and 24-hour means for PM_2.5_, PM_10_, and SO_2_ were obtained using the hourly concentrations based on the EPA’s National Ambient Air Quality Standard guidelines [28]. Finally, calculated measures from all 4 stations in Ahvaz were averaged to obtain the daily city-wide concentrations of pollutants. Because of the low percentage of missing data, imputation was not performed for either weather or air pollution data.

### Statistical analysis

Poisson additive models with overdispersion combined with the penalized DLNM were used to assess the association between air temperature and the daily number of deaths from CVD and respiratory diseases. The median of the daily mean temperature was used as the baseline value to calculate the relative risks (RRs). Day of the week, holidays, relative humidity, wind speed, and levels of air pollutants were controlled for as confounders in the model. To control for seasonal and long-term trends in the daily number of deaths, time was included in the model with a natural cubic spline function and 10 degrees of freedom (df) per year. To assess the heat and cold effects, the cumulative mortality risk was calculated at the 99th percentile relative to the 75th percentile and at the 1st percentile relative to the 25th percentile, respectively.

The daily mean temperature was included in the model as a penalized distributed lag non-linear term (characterized as a crossbasis matrix). Marginal P-spline smoothers with dimensions of 10 for both lag and exposure spaces were used, yielding a cross-basis matrix with 90 (10×9) parameters for the bidimensional space of lag and exposure. A double varying penalty with a second-order difference penalty was used along with a ridge penalty [7]. To incorporate the effects of cold (which have been shown to last for over 25 days [5]) and the harvesting effects, a maximum of 28 lag days was used for temperature.

Subgroup analyses were conducted to evaluate the effect modification by sex and age group. Patients were categorized into 3 groups by age (< 60, 60-74, and ≥ 75 years). To assess the modification by air pollutants of the effect of temperature on mortality, the level of each air pollutant was categorized into either greater than the median value or less than/equal to the median value. Then, an interaction term was generated between the cross-basis matrix and the strata of the air pollutant. Models were run separately for each interaction term corresponding to a single air pollutant while controlling for all other pollutants as well as day of the week, holiday status, relative humidity, and wind speed. The multivariate Wald test was used to test the statistical significance of differences between the overall temperature-mortality associations at the 2 levels of air pollution based on the coefficients of the cross-basis matrix of temperature [29]. Moreover, modifications of the heat and cold effects by air pollutants were assessed by evaluating the heat and cold effects at each of the 2 levels of air pollutants by lag 14 days and 28 days.

### Sensitivity analyses

Sensitivity analyses were performed to define the best model by changing the dimension of the marginal P-spline smoothers (4 to 10), the df (5 to 10 per year) for the time trend, and the maximum lag period for temperature (14 to 28 days). Based on the results, marginal P-spline smoothers with dimensions of 10 were used for both lag and exposure spaces, a df of 10 per year was used for the time trend, and the maximum lag period was set at 28 days, as these parameters produced models with the lowest restricted maximum likelihood. R version 3.6.1 (https://cran.r-project.org/bin/windows/base/old/3.6.1/) with the packages dlnm and mgcv were used to perform the analyses.

### Ethics statement

Ethical approval was obtained from the Shahid Beheshti University of Medical Sciences Ethics Committee (No. IR.SBMU.PHNS. REC.1397.111).

## RESULTS

During the study period, the recorded numbers of deaths from CVD and respiratory diseases were 5,639 and 1,030, respectively (males, 3,143 and 605; females, 2,496 and 425, respectively). The percentages of deaths by age group were 21.6%, 29.9%, and 48.3% from CVD and 32.0%, 25.1%, and 42.8% from respiratory diseases for individuals < 60 years, 60-74 years, and ≥ 75 years old, respectively. The characteristics of the variables used in the present study are shown in Table 1. The average daily mean temperature, relative humidity, and wind speed over the study period were 26.9°C, 41% and 2 m/s, respectively. The mean concentrations of PM_10_, PM_2.5_, CO, O_3_, SO_2_, and NO_2_ were 187 µg/m^3^, 57 µg/m^3^, 2,474 µg/m^3^, 74 µg/m^3^, 38 µg/m^3^, and 144 µg/m^3^, respectively. Relative humidity and wind speed had the lowest (0.1%) and CO had the highest (5.0%) proportion of missing values.

**Figure 1.**
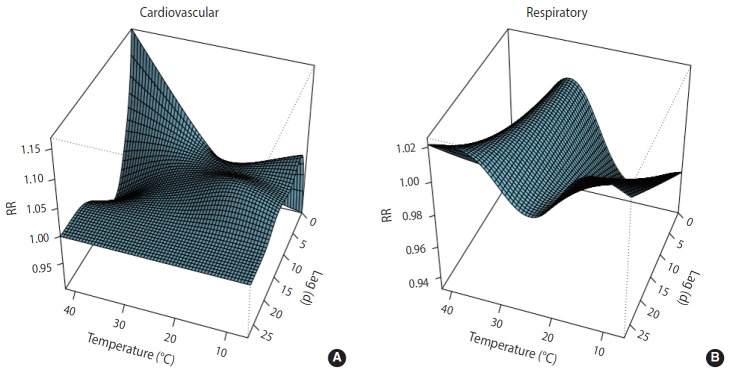
3D plot of the relationship between temperature and cardiovascular (A) and respiratory (B) mortality.

Along lag durations of 28 days, a non-linear relationship between temperature and number of deaths from CVD and respiratory diseases was revealed by the distributed non-linear lag surface (Figure 1).

The lag structures of cold and heat effects on cardiovascular mortality over 28 days are shown in Supplementary Material 1. High temperature had a weak and non-significant effect observed from the same day as exposure (lag 0) to 3 days after exposure; after this, a harvesting effect was observed until day 17. Cold temperature also had a weak and non-significant effect, which was delayed until lag 2 and lasted until lag 7. The cumulative effects of high and low temperatures on cardiovascular mortality over the 28 days are shown in Tables 2 and 3, respectively. The cumulative effect of high temperature increased by 10% until day 4, but gradually decreased afterward due to harvesting effects.

No significant heat or cold effects on respiratory mortality were observed (Supplementary Material 1). The cumulative effect of cold on respiratory mortality gradually increased from lag 0 days to lag 28 days so that by lag 28, the effect had increased by 34% (RR, 1.34; 95% confidence interval [CI], 0.44 to 4.08); however, this effect was not significant (Table 3).

In the subgroup analyses, no significant heat or cold effects on cardiovascular and respiratory mortality were seen among males or females. Among females, the cumulative effect of cold on cardiovascular and respiratory mortality steadily increased over the 28 days. The strongest effects were seen on day 28, with an RR of 2.56 (95% CI, 0.54 to 11.98) for respiratory mortality and an RR of 1.13 (95% CI, 0.77 to 1.66) for cardiovascular mortality, but these effects were not significant (Table 3).

With respect to age, no heat effect on cardiovascular mortality was observed among individuals < 60 years and 60-74 years old; however, an immediate effect was observed in the ≥ 75-year-old group. The strongest effect was seen on day 0 and was statistically significant (RR, 1.07; 95% CI, 1.00 to 1.16) and was followed by a harvesting effect from day 5 to day 19. The cold effect on cardiovascular mortality had a pattern that varied based on age group. An immediate effect was observed among individuals < 60 years old, with the strongest effect on day 0, and this effect gradually decreased over the following days and ended on day 13. No cold effect was noted among those 60-74 years old, but among those ≥ 75 years old, a cold effect was observed on day 2 and lasted 28 days (Supplementary Material 1). As for respiratory mortality, no heat effect was observed among any of the age groups. Moreover, no cold effect was observed among those 60-74 years or ≥ 75 years old; however, an immediate effect was observed among the < 60-year group, with the strongest effect on day 0. This effect lasted nonlinearly until day 25 (Supplementary Material 1).

Supplementary Material 2 shows the overall cumulative effects of temperature on cardiovascular and respiratory mortality. In the early days, the effect of higher temperatures on cardiovascular mortality was greater than that of lower temperatures, but at lag 14 days and later, the effect of lower temperatures was greater. This pattern can be explained by the delayed effects of lower temperatures and the harvesting effect observed with higher temperatures. The overall cumulative effects were similar on days 21 and 28, indicating a weak or non-existent effect of temperature after day 21. A similar pattern was seen for males. Among females, however, the effect of lower temperatures was greater than that of higher temperatures in almost all lag structures. Neither high nor low temperatures had a significant effect on respiratory mortality.

**Figure 2.**
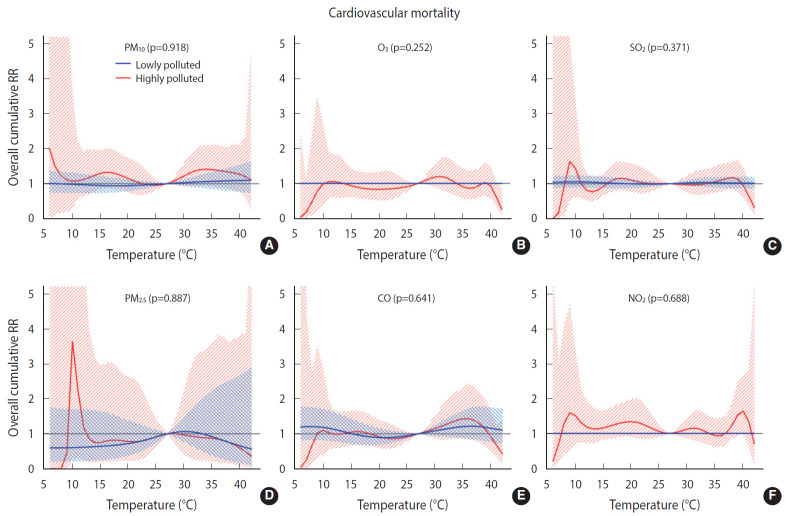
Overall cumulative temperature-cardiovascular mortality associations modified by air pollutants (A) particulate matter less than 10 µm (PM_10_), (B) ozone (O_3_), (C) sulfur dioxide (SO_2_), (D) particulate matter less than 2.5 µm (PM_2.5_), (E) carbon monoxide (CO), and, (F) nitrogen dioxide (NO_2_) (by lag1) with 95% confidence intervals. RR, relative risk.

**Figure 3.**
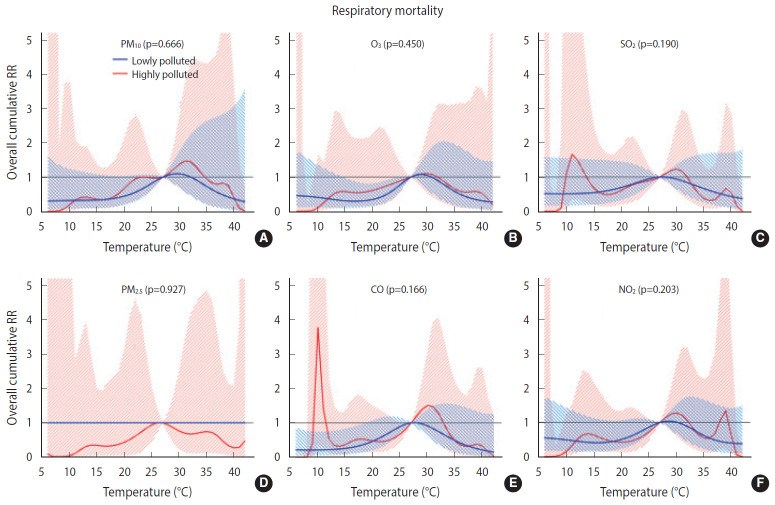
Overall cumulative temperature-respiratory mortality associations modified by air pollutants (A) particulate matter less than 10 µm (PM_10_), (B) ozone (O_3_), (C) sulfur dioxide (SO_2_), (D) particulate matter less than 2.5 µm (PM_2.5_), (E) carbon monoxide (CO), and, (F) nitrogen dioxide (NO_2_) (by lag1) with 95% confidence intervals. RR, relative risk.

Overall, we found no significant difference in the temperature-mortality association between the low and high pollution strata for either cardiovascular or respiratory mortality (Figures 2 and 3). No clear evidence was present for the modification of the cold or heat effects on CVD or respiratory disease by air pollutants (Table 4).

## DISCUSSION

Overall, the association of temperature with cardiovascular and respiratory mortality was non-linear. Heat effects on cardiovascular mortality were observed immediately, with the largest effect at lag 0, and cold effects appeared after 2 days and continued until day 7. In the subgroup analyses, high temperature was found to be significantly associated with an increased risk of mortality among the oldest individuals (≥ 75 years), and a stronger effect was seen in this group than in the other groups. Among those ≥ 75 years old, heat effects were observed immediately, but the cold effects were delayed. In individuals 60-74 years old, neither heat nor cold had a significant effect. Finally, among the youngest age group (< 60 years), no heat effect was seen, but a cold effect was observed on the day of exposure.

The effects of heat and cold were stronger among males than among females. Among males, as with the total population, the heat effects were observed immediately, with the strongest association on day 0 and a subsequent harvesting effect.

No significant association was observed between temperature and respiratory mortality, either overall or in the subgroup analyses by sex and age group. No heat effect on respiratory mortality was observed overall or in the sex and age subgroups, except in males, among which an effect was observed on days 0 to 2 followed by a harvesting effect from day 5 to day 13. In general, cold had a consistent weak effect on respiratory mortality over days 0 to 28. Cold effects were observed immediately, with the largest effect on day 0 and lasting until day 28 in females, but these effects were delayed in males. The cold effect was also much stronger in females than in males. Finally, the cold effect on respiratory mortality was much greater in the youngest group (< 60 years) than in either of the other groups, with the youngest group exhibiting an approximately 6-fold increase in the cumulative effect by day 28.

In general, in this study, we found that high temperature had an acute and short-term effect on mortality followed by a harvesting effect. We also found that cold temperature had a delayed effect on cardiovascular mortality. Similar lag structures have been found in a study performed in the city of Rasht, Iran [10] and in studies conducted in different countries [5,30,31].

Similar to the current study, other studies performed in Iran and other countries [5,10,32,33] have indicated that the effect of temperature on cardiovascular mortality is modified by age, with the elderly being more vulnerable to extreme heat than younger people. In the present report, subgroup analyses revealed that compared to older individuals, those in the youngest age group (< 60 years) are more vulnerable to cold effects, as was found in a study performed in Rasht, Iran [10]. Other studies on respiratory [34] and cardiovascular mortality [35] had similar results; however, these findings are inconsistent with those of the majority of previous studies [2,36-39]. Contrary to what has been reported in the literature (namely, that females are more vulnerable than males to heat effects), the current results showed the presence of a heat effect in males, but not in females. This inconsistency indicates that temperature-related mortality could vary based on the study setting and demographic characteristics, as demonstrated in previous studies [5,40]. These findings (a higher risk of mortality among younger individuals when exposed to cold and among males when exposed to heat) could be interpreted partially in the context of occupational exposure. A far higher percentage of the youngest subgroup than the oldest subgroup, as well as more males than females, work outdoors and are directly exposed to cold and heat.

To the best of the authors’ knowledge, this study is the first of its kind to investigate the modification of temperature effects by air pollutants on cardiovascular and respiratory mortality in Iran. Overall, the results revealed no interactive effects between temperature and air pollutants on cardiovascular and respiratory mortality. The interactive effect between temperature and air pollution is being studied worldwide with various modeling strategies. Previous studies have been focused mainly on the interactive effects of PM_10_ and O_3_, and much less so on the effects of NO_2_ and SO_2_. The findings are also highly inconsistent in this regard. For example, Ren et al. [41] reported that the effect of temperature on cardiovascular mortality was modified by PM_10_. In a study conducted by Chen et al. [15] in European urban areas, the effect of temperature on cardiovascular mortality was not significantly modified by PM_10_, PM_2.5_, or O_3_. In another study, PM_10_ was found to modify the effect of temperature on cardiovascular mortality but not on respiratory mortality. In addition, subgroup analyses revealed an effect modification for the elderly and for males, but not for those < 65 years of age or females [14]. Other studies have reported that heat effects were increased at high levels of O_3_ [42] and PM_10_ [43], whereas such an interactive effect has not been found for cold effects. In contrast, another study reported an interaction between PM_10_ and O_3_ and low temperatures on cardiovascular and respiratory mortality. In that study, no interaction was found between temperature and NO_2_ and SO_2_ [44]. Interestingly, in other studies, the effect modification by O_3_ was heterogeneous by area. For example, in a study conducted in the United States, effect modification by O_3_ on cardiovascular mortality was observed for northern but not for southern cities [45]. Likewise, in another study, which was conducted in 25 Italian cities and reported effect modifications by PM_10_ and O_3_ on natural mortality, effect modification by O_3_ was observed for northern cities but not for southern cities [46]. The findings of other studies have demonstrated an interactive effect between temperature and air pollution that is dependent upon age and sex [9,41].

The variation in findings between studies performed in different regions may be explained by a number of factors, such as differing levels of air pollutants and different cut-points used for stratification, the number and types of pollutants controlled for in the model, the demographic characteristics (such as age structure) of the population in the study area, ecological factors, the modeling strategy used, and the span of the study. Additionally, given the different temperature ranges of different regions and the fact that temperature effects are non-linear with the largest effects at extreme low and high temperatures (which vary by area), the lag structure seen for the temperature-mortality relationship could vary independently of other factors. For example, unlike the study performed in Rasht, Iran, and other studies [10,47], the current study demonstrated no cold effect on cardiovascular or respiratory mortality, likely due to the higher value of the low limit of mean temperature in this study compared to the other mentioned studies.

Changes in thermoregulatory and circulatory systems have been proposed as potential mechanisms to explain the increased risk of cardiovascular mortality as a result of exposure to extreme temperature [48]. Such exposure could lead to pathophysiological changes, such as increases in platelet count, blood viscosity, plasma fibrinogen level, red blood cell count, cholesterol level, sweating threshold, and blood pressure, all of which could exacerbate cardiopulmonary disease and lead to mortality [17,47]. Elderly people are more vulnerable than younger individuals to such changes because of poorer physiological adaptation.

One strength of the current study is that it is the first study to investigate the modification of temperature effect by air pollutants in Iran. Another strength is that a large number of air pollutants were considered. Moreover, a small proportion of data was missing. Unlike previous studies which used the linear or single-lagged temperature term to assess the effect modification by air pollutants, the distributed lag non-linear temperature term was used in the present study, which allowed non-linearity and lagged effects to be captured in both dimensions of association.

The current study also had some limitations. In this single-city study, the effect of temperature on mortality and modification of this effect by air pollutants were investigated only in Ahvaz, and the findings may not be generalizable to suburban and rural areas or to other cities. As in all ecological studies, exposure to temperature and air pollutants was assumed to be at the same level (measured as daily mean temperature and air pollutant level) for all people, which may have resulted in measurement error. Data on individual-level parameters, such as the use of air conditioning, activity patterns, and smoking status, were not available and may act as modifiers.

In conclusion, a weak, yet significant association exists between high temperature and cardiovascular mortality, but only in elderly people. Overall, we found no modification of the effect of ambient temperature on cardiovascular and respiratory mortality by air pollution.

## Figures and Tables

**Table 1.** Descriptive statistics for mortality, weather, and air pollution variables during the study period (from March 21, 2014 to March 20, 2018) in Ahvaz, Iran

Variables	Missing daily value (%)	Mean±SD	Percentile								
			Min	1	10	25	50	75	90	99	Max
Temperature (°C)	1.0	26.95±9.59	5.8	9.3	13.9	18.1	27.3	36.6	39	41.2	42.4
PM_10_ (µg/m^3^)	2.0	187.92±278.18	28.9	48.4	77.4	98.1	128.5	171.1	282.1	1521.7	3421.8
PM_2.5_ (µg/m^3^)	2.0	57.01±49.19	7.7	17.4	27.2	34.8	46.7	61.6	80.1	322.7	559.8
CO (µg/m^3^)	5.0	2,474.39±1,285.80	271.0	496.6	1,065.0	1,547.0	2,278.0	3,140.0	4,055.2	6,678.9	9,357.0
O_3_ (µg/m^3^)	3.0	74.67±44.20	7.0	16.0	31.0	41.2	65.0	101.5	128.0	223.0	471.0
SO_2_ (µg/m^3^)	3.0	38.88±26.87	0.9	6.9	16.3	22.2	32.3	47.3	68.4	131.2	342.7
NO_2_ (µg/m^3^)	3.0	144.32±132.83	0.0	17.5	36.1	59.4	104.9	181.3	298.8	709.7	925.1
Relative humidity (%)	0.1	41.58±17.56	9.1	15.0	21.0	27.1	38.6	53.9	67.0	85.9	92.3
Wind speed (m/s)	0.1	2.14±1.08	0.0	0.4	0.9	1.4	2.0	2.8	3.6	5.6	8.1

SD, standard deviation; Min, minimum; Max, maximum; PM_10_, particulate matter less than 10 μm in diameter; PM_2.5_, particulate matter less than 2.5 μm in diameter; CO, carbon monoxide; O_3_, ozone; SO_2_, sulfur dioxide; NO_2_, nitrogen dioxide.

**Table 2.** Cumulative effects of high temperature on cardiovascular and respiratory mortality (99th percentile of temperature [41.2°C] relative to 75th percentile [36.6°C]) during the study period (from March 21, 2014 to March 20, 2018) in Ahvaz, Iran

Lag (d)		Total	Male	Female	Age (yr)		
					<60	60-74	≥75
Cardiovascular mortality							
	Mean±SD (range)	3.8±2.1 (0-13)	2.1±1.5 (0-9)	1.7±1.3 (0-8)	0.8±0.9 (0-5)	1.1±1.1 (0-6)	1.8±1.4 (0-9)
	0	1.04 (0.98, 1.11)	1.02 (0.98, 1.07)	0.99 (0.97, 1.02)	1.06 (0.97, 1.15)	0.99 (0.96, 1.01)	1.07 (1.00, 1.16)
	0-3	1.10 (0.92, 1.32)	1.05 (0.95, 1.16)	0.99 (0.90, 1.08)	1.01 (0.82, 1.23)	0.97 (0.88, 1.07)	1.14 (0.92, 1.41)
	0-5	1.08 (0.85, 1.38)	1.05 (0.92, 1.19)	0.98 (0.86, 1.12)	1.01 (0.77, 1.32)	0.96 (0.84, 1.10)	1.11 (0.83, 1.48)
	0-7	1.03 (0.77, 1.39)	1.03 (0.89, 1.20)	0.97 (0.82, 1.14)	1.01 (0.74, 1.39)	0.95 (0.80, 1.13)	1.05 (0.73, 1.49)
	0-14	0.85 (0.55, 1.33)	0.98 (0.78, 1.21)	0.94 (0.73, 1.21)	1.02 (0.68, 1.55)	0.92 (0.70, 1.22)	0.80 (0.46, 1.38)
	0-21	0.82 (0.50, 1.35)	0.93 (0.72, 1.21)	0.93 (0.69, 1.25)	1.03 (0.67, 1.58)	0.91 (0.64, 1.28)	0.71 (0.35, 1.41)
	0-28	0.82 (0.50, 1.35)	0.91 (0.69, 1.21)	0.93 (0.68, 1.28)	1.03 (0.67, 1.58)	0.90 (0.62, 1.30)	0.72 (0.32, 1.58)
Respiratory mortality							
	Mean±SD (range)	0.7±0.9 (0-5)	0.4±0.6 (0-4)	0.2±0.5 (0-4)	0.2±0.5 (0-5)	0.1±0.4 (0-3)	0.3±0.5 (0-4)
	0	0.96 (0.88, 1.04)	1.03 (0.84, 1.26)	0.94 (0.85, 1.04)	0.84 (0.57, 1.23)	0.96 (0.73, 1.28)	0.98 (0.89, 1.09)
	0-3	0.87 (0.63, 1.19)	1.07 (0.56, 2.01)	0.80 (0.56, 1.15)	0.58 (0.19, 1.78)	0.88 (0.30, 2.55)	0.95 (0.65, 1.40)
	0-5	0.82 (0.52, 1.29)	1.05 (0.44, 2.48)	0.72 (0.43, 1.20)	0.40 (0.09, 1.77)	0.84 (0.18, 3.86)	0.94 (0.54, 1.63)
	0-7	0.77 (0.43, 1.38)	1.00 (0.35, 2.88)	0.65 (0.34, 1.24)	0.23 (0.03, 1.46)	0.80 (0.11, 5.63)	0.93 (0.46, 1.86)
	0-14	0.66 (0.26, 1.68)	0.88 (0.17, 4.44)	0.49 (0.18, 1.36)	0.04 (0.00, 0.81)	0.70 (0.03, 15.87)	0.90 (0.30, 2.71)
	0-21	0.61 (0.19, 1.90)	0.93 (0.12, 6.83)	0.43 (0.12, 1.53)	0.03 (0.00, 1.04)	0.64 (0.01, 29.05)	0.90 (0.23, 3.42)
	0-28	0.59 (0.17, 2.03)	1.00 (0.11, 8.91)	0.44 (0.11, 1.82)	0.02 (0.00, 1.29)	0.62 (0.01, 35.67)	0.91 (0.21, 3.85)

Values are presented as relative risk (95% confidence interval).

**Table 3.** Cumulative effects of low temperature on cardiovascular and respiratory mortality (1st percentile of temperature [9.3°C] relative to 25th percentile [18.1°C]) during the study period (from March 21, 2014 to March 20, 2018) in Ahvaz, Iran

Lag (d)		Total	Male	Female	Age (yr)		
					<60	60-74	≥75
Cardiovascular mortality							
	0	0.97 (0.89, 1.06)	0.94 (0.87, 1.02)	1.01 (0.97, 1.05)	1.06 (0.97, 1.15)	0.99 (0.97, 1.02)	0.94 (0.84, 1.05)
	0-3	1.03 (0.85, 1.24)	0.90 (0.75, 1.08)	1.04 (0.92, 1.18)	1.19 (0.94, 1.51)	0.99 (0.89, 1.10)	1.01 (0.78, 1.28)
	0-5	1.08 (0.86, 1.36)	0.91 (0.72, 1.15)	1.06 (0.90, 1.26)	1.26 (0.92, 1.71)	0.99 (0.85, 1.15)	1.08 (0.79, 1.47)
	0-7	1.11 (0.85, 1.45)	0.93 (0.71, 1.22)	1.07 (0.87, 1.32)	1.30 (0.90, 1.88)	0.99 (0.81, 1.20)	1.13 (0.79, 1.63)
	0-14	1.09 (0.75, 1.59)	1.03 (0.70, 1.52)	1.09 (0.80, 1.48)	1.38 (0.86, 2.21)	0.99 (0.73, 1.35)	1.23 (0.73, 2.08)
	0-21	1.10 (0.72, 1.70)	1.12 (0.70, 1.79)	1.11 (0.77, 1.59)	1.39 (0.84, 2.28)	1.00 (0.68, 1.46)	1.45 (0.76, 2.79)
	0-28	1.10 (0.72, 1.70)	1.16 (0.70, 1.93)	1.13 (0.77, 1.66)	1.39 (0.84, 2.28)	1.01 (0.67, 1.54)	1.61 (0.76, 3.39)
Respiratory mortality							
	0	1.01 (0.94, 1.07)	0.86 (0.69, 1.09)	1.08 (0.95, 1.24)	1.18 (0.75, 1.84)	1.00 (0.84, 1.18)	0.98 (0.89, 1.09)
	0-3	1.03 (0.80, 1.33)	0.75 (0.42, 1.33)	1.34 (0.87, 2.07)	1.49 (0.59, 3.78)	1.01 (0.53, 1.93)	0.95 (0.66, 1.37)
	0-5	1.05 (0.72, 1.51)	0.78 (0.38, 1.58)	1.51 (0.83, 2.74)	1.97 (0.64, 6.05)	1.02 (0.40, 2.58)	0.93 (0.56, 1.53)
	0-7	1.06 (0.67, 1.70)	0.80 (0.35, 1.84)	1.68 (0.80, 3.49)	2.61 (0.70, 9.64)	1.03 (0.31, 3.38)	0.91 (0.48, 1.69)
	0-14	1.14 (0.53, 2.43)	0.76 (0.23, 2.54)	2.07 (0.67, 6.37)	3.28 (0.47, 22.51)	1.07 (0.15, 7.29)	0.85 (0.32, 2.24)
	0-21	1.23 (0.47, 3.21)	1.03 (0.23, 4.69)	2.35 (0.58, 9.46)	7.25 (0.62, 83.68)	1.13 (0.10, 12.32)	0.85 (0.25, 2.88)
	0-28	1.34 (0.44, 4.08)	1.30 (0.22, 7.55)	2.56 (0.54, 11.98)	6.31 (0.34, 115.4)	1.21 (0.08, 17.46)	0.94 (0.24, 3.66)

Values are presented as relative risk (95% confidence interval).

**Table 4.** Air pollutant-stratified associations of heat and cold with cardiovascular and respiratory mortality using multiple cut-off points during the study period (from March 21, 2014 to March 20, 2018) in Ahvaz, Iran

Pollutant		Cardiovascular mortality				Respiratory mortality			
		Lag 14 d		Lag 28 d		Lag 14 d		Lag 28 d	
		Heat	Cold	Heat	Cold	Heat	Cold	Heat	Cold
CO (µg/m^3^)	Low	1.04 (0.87, 1.23)	0.99 (0.79, 1.25)	1.05 (0.84, 1.30)	1.01 (0.74, 1.33)	0.81 (0.28, 2.36)	0.68 (0.16, 2.81)	0.78 (0.20, 3.02)	0.61 (0.10, 3.72)
	High	1.01 (0.87, 1.16)	1.03 (0.85, 1.26)	1.01 (0.83, 1.21)	1.04 (0.81, 1.34)	0.82 (0.67, 0.99)	1.46 (1.01, 2.09)	0.77 (0.60, 0.98)	1.63 (1.02, 2.61)
O_3_ (µg/m^3^)	Low	1.00 (0.99, 1.00)	0.99 (1.00,1.00)	1.00 (0.99, 1.00)	0.99 (0.99, 1.00)	0.99 (0.99, 1.00)	0.99 (0.99, 1.00)	0.99 (0.99, 1.00)	0.99 (0.99, 1.00)
	High	0.98 (0.94, 1.02)	1.03 (0.95, 1.11)	0.98 (0.94, 1.02)	1.03 (0.94, 1.12)	1.10 (0.81, 1.50)	0.90 (0.57, 1.40)	1.09 (0.78, 1.53)	0.89 (0.55, 1.44)
SO_2_ (µg/m^3^)	Low	0.99 (0.99, 1.00)	0.99 (0.99, 1.00)	0.99 (0.99, 1.00)	0.99 (0.99, 1.00)	1.00 (0.99, 1.00)	0.99 (0.99, 1.00)	1.00 (0.99, 1.00)	0.99 (0.99, 1.00)
	High	1.11 (0.96, 1.28)	0.82 (0.63, 1.06)	1.18 (0.95, 1.47)	0.79 (0.55, 1.12)	0.92 (0.79, 1.08)	1.15 (0.86, 1.55)	0.92 (0.78, 1.08)	1.16 (0.85, 1.58)
NO_2_ (µg/m^3^)	Low	0.97 (0.92, 1.03)	1.03 (0.94, 1.14)	0.97 (0.92, 1.03)	1.04 (0.93, 1.15)	1.00 (0.99, 1.01)	1.00 (0.99, 1.01)	1.00 (0.99, 1.01)	1.00 (0.99, 1.01)
	High	0.99 (0.97, 1.02)	1.00 (0.96, 1.04)	0.99 (0.96, 1.02)	1.00 (0.95, 1.05)	0.99 (0.99, 1.00)	1.00 (0.99, 1.00)	0.99 (0.99, 1.00)	1.00 (0.99, 1.01)
PM_10_ (µg/m^3^)	Low	0.85 (0.55, 1.33)	1.09 (0.75, 1.59)	0.82 (0.50, 1.35)	1.11 (0.72, 1.70)	0.66 (0.26, 1.68)	1.14 (0.53, 2.43)	0.59 (0.17, 2.03)	1.34 (0.44, 4.08)
	High	0.99 (0.97, 1.02)	1.00 (0.96, 1.04)	0.99 (0.96, 1.02)	1.00 (0.95, 1.05)	0.84 (0.56, 1.25)	0.97 (0.60, 1.55)	0.83 (0.54, 1.27)	0.96 (0.58, 1.61)
PM_2.5_ (µg/m^3^)	Low	1.00 (0.97, 1.03)	0.99 (0.94, 1.04)	1.00 (0.97, 1.03)	0.99 (0.94, 1.04)	1.00 (0.99, 1.01)	0.99 (0.98, 1.01)	0.99 (0.99, 1.01)	0.99 (0.98, 1.01)
	High	0.98 (0.76, 1.26)	1.01 (0.72, 1.42)	0.98 (0.76, 1.27)	1.01 (0.71, 1.44)	0.65 (0.23, 1.85)	0.72 (0.17, 3.02)	0.58 (0.15, 2.16)	0.67 (0.10, 4.13)

Values are presented as relative risk (95% confidence interval). CO, carbon monoxide; O_3_, ozone; SO_2_, sulfur dioxide; NO_2_, nitrogen dioxide; PM_10_, particulate matter less than 10 μm in diameter; PM_2.5_, particulate matter less than 2.5 μm in diameter.
