# Supplementary material for: Modification of the effect of ambient air temperature on cardiovascular and respiratory mortality by air pollution in Ahvaz, Iran
Source: Epidemiol Health. 2020 Jul 18;42:e2020053. doi: 10.4178/epih.e2020053 (PMC7871149; doi:10.4178/epih.e2020053)
Supplement: Supplementary file 1 [file epih-42-e2020053-suppl.docx]

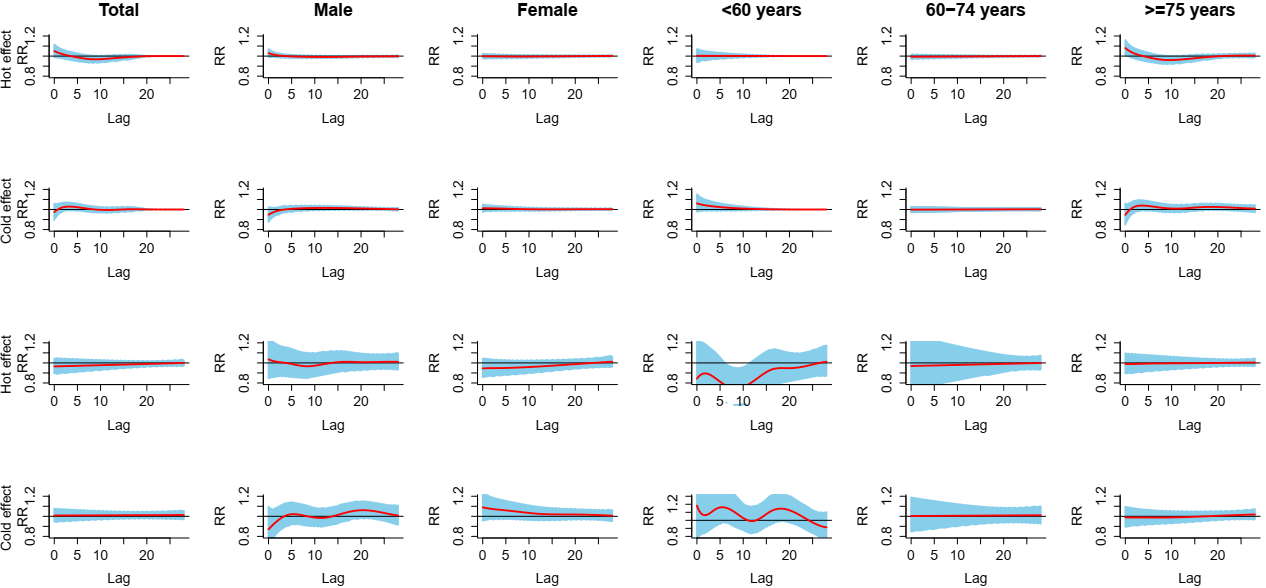


**Supple­mentary Material 1:** The estimated hot effects (99th percentile of temperature (41.2°C) relative to 75th percentile of temperature (36.6°C)) and cold effects (1st percentile of temperature (9.3°C) relative to 25th percentile of temperature (18.1°C)) of mean temperature on cardiovascular (row 1 and 2) and respiratory (row 3 and 4) mortality along the lag days.


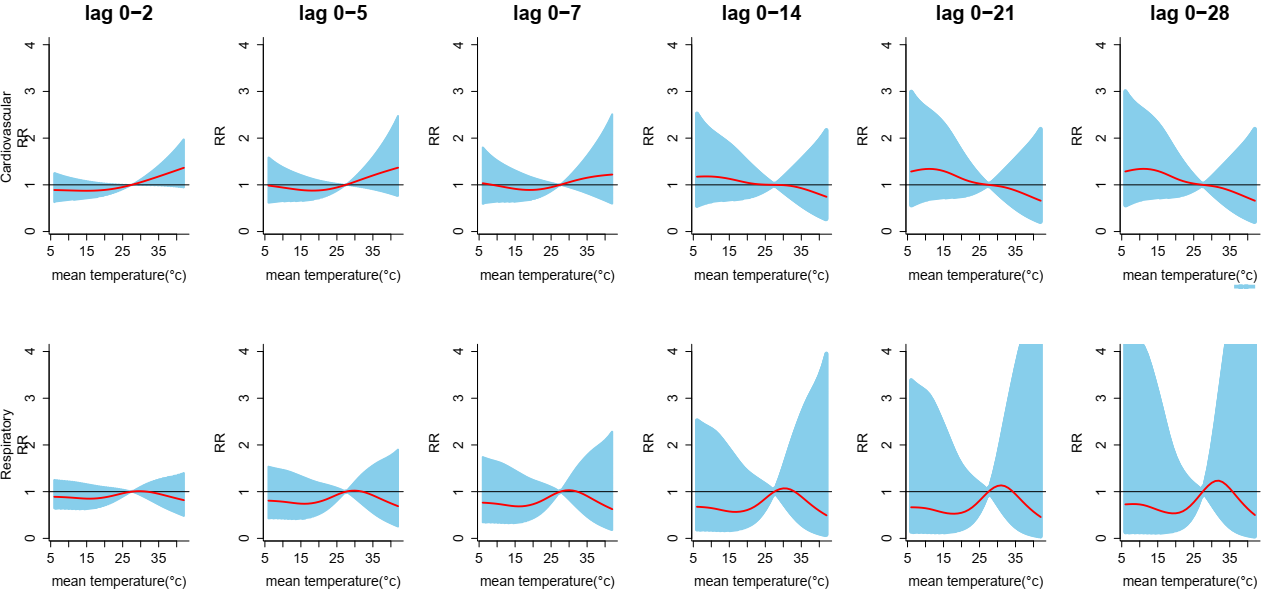


**Supple­mentary Material 2:** The overall cumulative temperature-mortality associations with 95% Cis in different lag structures. The reference value was median temperature (27.3°C).
